# Rewiring of Memory Circuits: Connecting Adult Newborn Neurons With the Help of Microglia

**DOI:** 10.3389/fcell.2019.00024

**Published:** 2019-03-05

**Authors:** Noelia Rodríguez-Iglesias, Amanda Sierra, Jorge Valero

**Affiliations:** ^1^Laboratory of Glial Cell Biology, Achucarro Basque Center for Neuroscience, Leioa, Spain; ^2^Department of Neuroscience, University of the Basque Country UPV/EHU, Leioa, Spain; ^3^Ikerbasque Foundation, Bilbao, Spain

**Keywords:** adult hippocampal neurogenesis, hippocampal circuit, memory, microglia, rewiring

## Abstract

New neurons are continuously generated from stem cells and integrated into the adult hippocampal circuitry, contributing to memory function. Several environmental, cellular, and molecular factors regulate the formation of new neurons, but the mechanisms that govern their incorporation into memory circuits are less explored. Herein we will focus on microglia, the resident immune cells of the CNS, which modulate the production of new neurons in the adult hippocampus and are also well suited to participate in their circuit integration. Microglia may contribute to the refinement of brain circuits during development and exert a role in physiological and pathological conditions by regulating axonal and dendritic growth; promoting the formation, elimination, and relocation of synapses; modulating excitatory synaptic maturation; and participating in functional synaptic plasticity. Importantly, microglia are able to sense subtle changes in their environment and may use this information to differently modulate hippocampal wiring, ultimately impacting on memory function. Deciphering the role of microglia in hippocampal circuitry constant rewiring will help to better understand the influence of microglia on memory function.

## Introduction

The generation of hippocampal neurons during adulthood has been implicated in critical brain functions, such as memory formation, and pathological conditions, including mood disorders. Therefore, a strong research effort has been put into determining the factors controlling the integration of newly generated neurons into the hippocampus during adulthood, and their impact on brain function. Different memory related functions have been attributed to the new neurons that integrate into the adult rodent hippocampus, which include pattern separation, temporal codification, and memory clearance (Baptista and Andrade, 2018). The persistence of hippocampal neurogenesis through adulthood in rodents is well established (Ming and Song, 2011), although it decreases exponentially over time (Beccari et al., 2017). In contrast, data on human neurogenesis are more conflicting, and there are recent reports to support either that it persists across human lifespan (Spalding et al., 2013; Boldrini et al., 2018) or, on the contrary, that it is limited to childhood (Sorrells et al., 2018). In any case, adult neurogenesis plays a more critical role during infancy, contributing to the codification and integration of early life experiences, and thus affecting the adaptability and function of the central nervous system (CNS) during adult life (Kempermann et al., 2018).

Adult hippocampal neurogenesis is tightly controlled by both cell-autonomous mechanisms as well as by the interaction with the molecular and cellular niche. In this review, we will focus on the contribution of microglia, the resident immune cells of the CNS, to the incorporation of adult generated neurons into hippocampal memory circuits. Microglia are especially well equipped to sense changes in the brain parenchyma and to interact with other cell types such as neurons and astrocytes (Kettenmann et al., 2011), and thus they are good candidates to act as mediators of the adaptive incorporation of newly generated neurons into hippocampal circuits, although few studies have addressed the role of microglia on neurogenesis directly (Valero et al., 2016, 2017). First, we will describe the main characteristics of hippocampal adult neurogenesis in rodents and how newborn cells incorporate into hippocampal circuitry. Then we will briefly introduce microglia and summarize their main functions in different contexts, such as development, adulthood or pathology, which may be relevant for the integration of new generated neurons into memory circuits. Finally, we will discuss evidence that suggest that microglia effectively play a role in neurite growth as well as in the structural and functional maturation of newborn neurons.

## Adult Hippocampal Neurogenesis and Its Regulation

Neurogenesis persists during adulthood in many species from invertebrates to vertebrates, including mammals (Sullivan et al., 2007; Bonfanti and Amrein, 2018), although newly generated neurons are fewer than those generated in embryonic stages and restricted to specific areas of the brain. Adult neurogenesis is initiated by adult neural stem cells, which give rise to functional neurons in the adult CNS (Ming and Song, 2011). Neural stem cells are mainly restricted to two regions of the mammalian adult brain, known as adult neurogenic niches: the subventricular zone of the lateral ventricles, and the subgranular zone (SGZ) of the hippocampal dentate gyrus (DG) (Altman, 1962; Altman and Das, 1965; Eriksson et al., 1998). In addition, adult neurogenesis has been described in other regions of the brain, such as the hypothalamus in rodents (Sousa-Ferreira et al., 2013) and the striatum in humans (Ernst et al., 2014). Here we will focus on the formation of new granular neurons in the SGZ of rodents, i.e., adult hippocampal neurogenesis.

**FIGURE 1:**
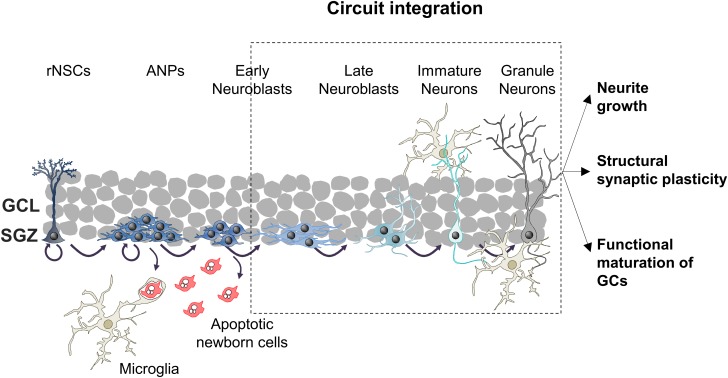
Schematic drawing of the neurogenic cascade and the hypothetical roles of microglia on adult neurogenesis. ANPs, amplifying neuroprogenitors; GCs, granule cells; GCL, granular cell layer; rNSCs, radial neural stem cells; SGZ, subgranular zone.

Adult hippocampal neurogenesis encompasses sequential cellular stages known as the neurogenic cascade (Figure 1). The generation of new neurons begins with radial neural stem cells (rNSCs, also known as type 1 cells), which usually divide asymmetrically giving rise to a copy of themselves and an amplifying neuroprogenitor (ANP, also known as type 2 cells) (Bonaguidi et al., 2011; Encinas et al., 2011). ANPs increase their pool rapidly through symmetric divisions and differentiate into neuron-committed cells called neuroblasts. However, not all ANPs and neuroblasts survive, as nearly the 80–90% of newly generated cells die through apoptosis (Sierra et al., 2010; Beccari et al., 2017). The surviving neuroblasts continue differentiating into immature neurons, and they integrate in pre-existing hippocampal circuitries finally becoming mature granule cells (GCs), the most abundant type of excitatory neuron in the DG (Kempermann et al., 2015). The maturation of adult-born granule neurons lasts around 8 weeks (Toda et al., 2018), and it will be described in detail in the next section.

The above described neurogenic cascade is susceptible of modulation at different levels: (1) proliferation of rNSCs and ANPs; (2) survival of ANPs and neuroblasts; (3) differentiation into neurons; and (4) integration of adult-born GCs (Kempermann et al., 2004; Valero et al., 2016, 2017). These steps are regulated by a tightly controlled program that encompasses intrinsic (i.e., signaling cascades and transcription factors), as well as extrinsic factors. Among these later ones stand out circulating hormones and peptides (such as cortisol, growth factors, or inflammatory mediators), extracellular matrix composition, and neighboring cells that compose the cellular niche: adult neurons, astrocytes, blood vessels and microglia (Sierra et al., 2010; Ming and Song, 2011; Valero et al., 2012, 2016, 2017; Aimone et al., 2014). In this review we will specifically focus on how microglia modulates the formation of adult-born GCs exerting their functions on the different steps of the neurogenic cascade, especially on the integration of the new neurons to the hippocampal circuitry (Figure 1).

## GCs and the Hippocampal Circuitry

### GCs Connection to the Tri-Synaptic Circuit

**FIGURE 2:**
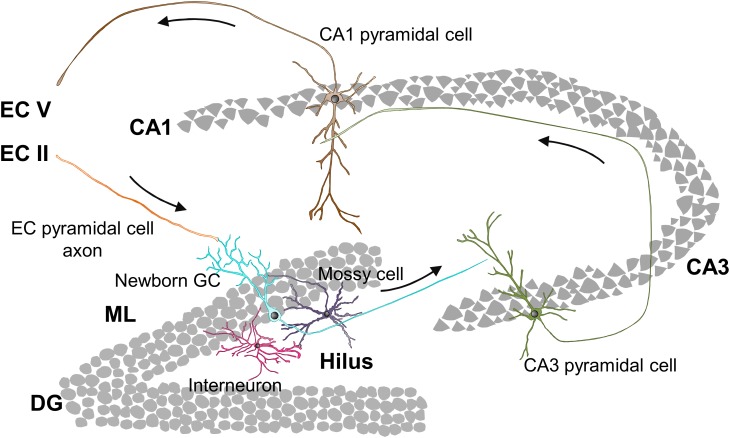
Hippocampal tri-synaptic circuit. CA1, Cornu Ammonis subfield 1; CA3, Cornu Ammonis subfield 3; DG, dentate gyrus; EC, entorhinal cortex; GC, granule cell; ML, molecular layer.

The hippocampal connectivity is characterized by the well-known tri-synaptic circuit, in which pyramidal cells located in layer II of the entorhinal cortex (EC) connect to DG GCs, which send their output signal to the Cornu Ammonis subfield 3 (CA3). Then, the signal runs from CA3 to CA1 pyramidal neurons and from CA1 back to neurons of the EC layer V (Figure 2). Nonetheless, hippocampal connectivity is more complex and includes direct connections from EC to CA3 and CA1 (Amaral et al., 2007), as well as direct inputs from the perirrhinal cortex (PER) and back projections from CA to the DG (Binicewicz et al., 2016). Within the tri-syanptic circuit, both developmentally and adult generated GCs have similar connectivity: they extend their dendrites into the molecular layer (ML), where they receive glutamatergic synaptic inputs from PER and EC axons; and send their axons (mossy fibers) through the polymorph layer (hilus) to CA3, where they establish highly efficient excitatory multisynaptic contacts with CA3 pyramidal neurons (Toni et al., 2008; Vivar et al., 2012). GCs mossy fibers present axonal boutons, which envelop and contact protuberances (thorny excrescences) from CA3 dendrites which provide multiple synaptic contact regions increasing synaptic efficacy (Faulkner et al., 2008; Toni et al., 2008). In addition to forming the tri-synaptic circuit, GCs are also connected to the local DG circuit, which contributes to the refinement of the information passed to CA3 (Figure 2).

### GCs Connection to the Local DG Circuitry

DG local circuits mainly consist in feedback connections between GCs and interneurons. GCs excite GABAergic hilar and CA3 interneurons, which in turn inhibit GCs (feedback inhibition) and CA3 pyramidal cells (feedforward inhibition) (McAvoy et al., 2015; Prince et al., 2016). Furthermore, GCs provide excitatory inputs to glutamatergic interneurons residing in the hilus (mossy cells), which play a dual role as they directly excite other GCs in the ipsi- and contralateral DG, but also indirectly inhibit GCs by acting on inhibitory interneurons (Scharfman, 2016). This internal excitatory/inhibitory system plays a crucial role in the processing of the input signal, in the refinement of the output sent to CA3, and in the maturation of adult-born GCs, as we will discuss in the following sections.

## Integration of Adult Newborn Neurons Into the Hippocampal Circuitry

Given the connectivity of GCs, it seems obvious that the integration of newborn neurons into the mature circuit is a complex and highly coordinated process that involves a series of morphological and functional stages. Importantly, the appearance of certain characteristics, such as the maximum complexity of the dendritic tree or number of dendritic spines, may be delayed in older animals and affected by life related factors such as voluntary exercise (Trinchero et al., 2017). In this section we will review the different stages followed by newborn GCs during their maturation and integration into the hippocampal circuit in rodents (in mice, unless otherwise specified), how these stages affect hippocampal function, and summarize the factors that regulate this process.

### Stages of GCs Maturation

**FIGURE 3:**
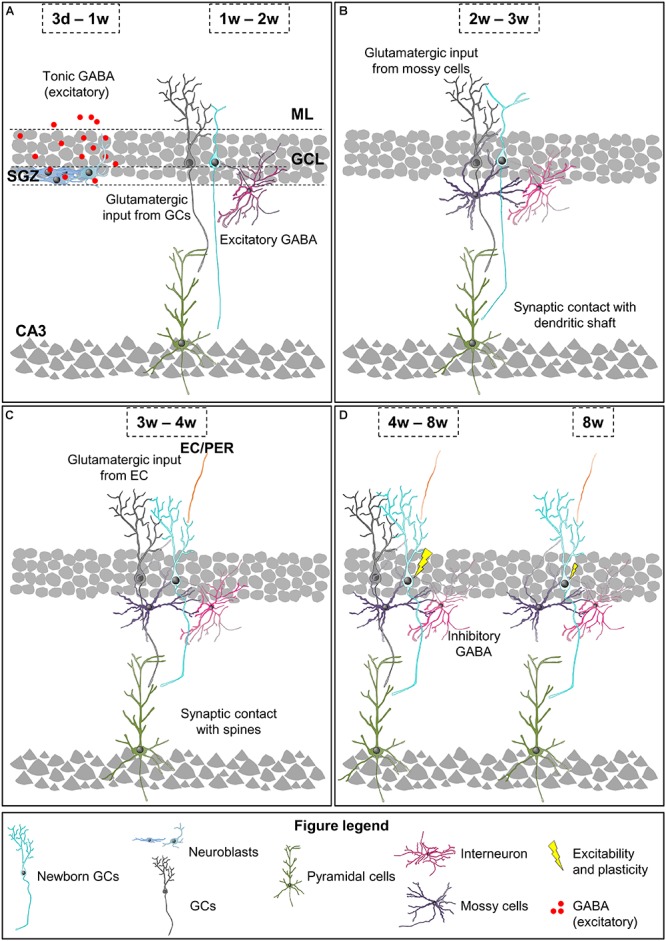
Integration stages of adult newborn neurons into the hippocampal circuitry. **(A)** 3 days to 1-week-old neuroblasts sense tonic GABA, which is excitatory. 1–2 weeks after their birth, newborn GCs receive their first glutamatergic input from mature GCs and they are contacted by GABAergic interneurons, while their axons reach the CA3. **(B)** 2–3-week-old newborn GCs receive glutamatergic input from mossy cells and they establish synaptic contacts with the dendritic shafts of CA3 pyramidal cells. **(C)** 3–4-week-old newborn GCs receive glutamatergic inputs from EC and PER, and their axons establish synaptic contacts with dendritic spines of CA3 pyramidal cells. **(D)** 4–8-week-old are fully integrated into the hippocampal circuitry, and show increased excitability and enhanced synaptic plasticity. 8-week-old newborn GCs are fully mature neurons and integrated into the hippocampal memory circuit. EC, entorhinal cortex; GC, granule cell; GCL, granule cell layer; ML, molecular layer; PER, perirhinal cortex; SGZ, subgranular zone.

At 3 days to 1 week newborn cells have already differentiated into early neuroblasts but are still located at the SGZ and present short processes with no synaptic contacts (Figure 3A; Esposito, 2005; Zhao et al., 2006). The first neurotransmitter sensed by these young cells is non-synaptic, tonic GABA (γ-aminobutyric acid), the most abundant inhibitory neurotransmitter in the CNS (Tozuka et al., 2005; Ge et al., 2006; Song et al., 2013). At this stage and up to 3 weeks after cell birth, similarly to what happens during hippocampal development, GABA is excitatory and depolarizes maturing GCs due to their high expression of the sodium-potassium-chloride cotransporter NKCC1, which maintains high levels of intracellular chloride (Tozuka et al., 2005; Ge et al., 2006; Campbell et al., 2010; Spampanato et al., 2012). GABA sensing from new GCs at this stage is crucial for their differentiation and survival (Tozuka et al., 2005; Ge et al., 2006; Song et al., 2013).

One to two weeks after their birth, the dendritic tree of newborn GCs reaches the ML and their axons CA3 (Figure 3A). Data about the synaptic contacts they receive are contradictory. Initially, it was shown that at this time point maturing GCs only received synaptic contacts from GABAergic interneurons (Overstreet-Wadiche et al., 2005; Ge et al., 2006; Jagasia et al., 2009; Song et al., 2013; Alvarez et al., 2016). However, a more recent study revealed contacts from glutamatergic, GABAergic, and cholinergic neurons in 1-week old GCs (Sah et al., 2017). At this time point, synaptic GABA is necessary for the morphological maturation, synaptic integration, and survival of newly generated neurons (Ge et al., 2006; Jagasia et al., 2009; Song et al., 2013; Alvarez et al., 2016). Specifically, during a narrow temporal window from 9 to 11 days after their birth the activation of GABAergic parvalbumin interneurons by mature GCs is required for the increase in the complexity of dendrites and number of spines induced by environmental enrichment (Alvarez et al., 2016). The axons of maturing GCs reach the stratum lucidum of CA3 and CA2 1 and 2 weeks after their birth, respectively (Zhao et al., 2006; Faulkner et al., 2008; Llorens-Martín et al., 2015). The initial excitatory synaptic contacts from newborn GCs onto pyramidal cells of the CA3 occur 1 week after their birth, while connectivity into the CA2 has not been analyzed (Ide et al., 2008; Toni et al., 2008). Therefore, by their second week after birth a few maturing GCs are already wired into the hippocampal circuit as they receive excitatory inputs in the DG and contact with CA3 pyramidal cells.

Two to three week-old newborn GCs establish a first circuit, as they receive excitatory inputs in the DG, send projections to CA2 and CA3 pyramidal cells, and are able to fire their first action potentials (Figure 3B). The dendritic tree of maturing GCs reaches the inner ML 2 weeks after their birth. Several studies describe that the unique excitatory synaptic contact received by 2 week old GCs is established with hilar mossy cells (Esposito, 2005; Ge et al., 2006; Zhao et al., 2006; Deshpande et al., 2013; Chancey et al., 2014). However, a recent study indicates that other excitatory intrahippocampal neurons, such as mature GCs and CA pyramidal cells, already innervate maturing GCs (Sah et al., 2017). Nevertheless, dendritic spines on newborn GCs are not visible until day 16 after their birth (Zhao et al., 2006). In addition to give a direct excitatory input to maturing GCs, mossy cells also provide indirect depolarization to new GCs through activation of GABAergic interneurons, which combined with the weak excitatory input may help maturing GCs to develop their first action potentials (Chancey et al., 2014). Importantly, during the first 2–3 weeks after cell birth a moderated GABA input is able to induce action potentials in young GCs when paired to the weak glutamatergic input (Heigele et al., 2016). Indeed, at this stage, GABA depolarization is required for synapse unsilencing through activation of NMDA receptors and subsequent recruitment of AMPA receptors to synapses (Chancey et al., 2013). These data suggest that GABAergic mediated excitation of 2–3 week-old GCs may occur during regular hippocampal activity, and that it depends on glutamatergic input. Importantly, as previously indicated, GABAergic input is still essential for the survival of maturing GCs, which may be due to the relevant role of GABA on the firing of the first action potentials. Furthermore, NMDA receptor expression and activation is also crucial for the survival of maturing GCs specifically between weeks 2 and 3 after their birth (Tashiro et al., 2007); suggesting that, at this time point, both inhibitory and excitatory inputs support cell survival. At 17 days after birth, the mossy fiber boutons from newborn neurons form synapses mainly with the dendritic shaft of CA3 pyramidal neurons and dendrites from hilar and CA3 interneurons (Toni et al., 2008; Gu et al., 2012). Hence, at 3 weeks after their birth the majority of maturing GCs receives excitatory inputs in the ML and are connected to CA3 pyramidal cells.

Three to four week-old newborn GCs receive glutamatergic synaptic contacts from PER and EC axons and increase their synapses with CA3 pyramidal cell dendrites (Figure 3C). At 3 weeks after birth, when the dendritic tree of new neurons has already reached the outer ML and their length and complexity peak, axons from the PER, and EC establish synaptic contacts with new born GCs (Esposito, 2005; Zhao et al., 2006; Vivar et al., 2012; Deshpande et al., 2013; Trinchero et al., 2017). Importantly, at this stage, dendritic spines are few and highly unstable (Toni et al., 2007). Accordingly, glutamatergic inputs are weak and do not induce action potentials but, as previously indicated, may facilitate its generation when combined with moderate depolarizing GABAergic inputs (Heigele et al., 2016). Meanwhile, in CA3 layer, the axonal boutons from newborn GCs already contact with dendritic spines or thorny excrescences from CA3 pyramidal neurons (Faulkner et al., 2008; Toni et al., 2008). The size of the boutons, number of spines they envelop, and active synaptic contacts increase until the fourth week after cell birth (Faulkner et al., 2008). Therefore, 4 week-old GCs are already connecting EC and CA3, thus forming part of the classical tri-synaptic hippocampal circuitry.

Four to eight weeks after their birth newly generated GCs are fully integrated into the adult hippocampal circuitry but still have special electrophysiological features compared to fully mature GCs: increased excitability and enhanced synaptic plasticity (Schmidt-Hieber et al., 2004; Ge et al., 2007; Mongiat et al., 2009; Gu et al., 2012; Figure 3D). At this time point, GABA action onto new GCs is already inhibitory, presumably due to a decrease in the chloride importer NKCC1 and an increase in the chloride exporter KCC2 expression (Ge et al., 2006). Importantly, 4–6 week-old GCs are less influenced by feedback inhibition than mature GCs, which contributes to their higher excitability (Marín-Burgin et al., 2012; Dieni et al., 2013). Moreover, the presence of the GluN2B (NR2B) subunit of the NMDA receptor and the T-type calcium channel in these new GCs provides enhanced synaptic plasticity (Schmidt-Hieber et al., 2004; Ge et al., 2007; Gu et al., 2012). All these characteristics favor the activation and subsequent recruitment of 4 week-old newborn GCs to memory engrams, suggesting a relevant involvement in memory function. Indeed, silencing 4 week-old GCs, but not 2 or 8 week-old GCs, interferes with memory retrieval indicating that, at this stage, newborn GCs store relevant memory information (Gu et al., 2012). The density of GC dendritic spines reaches a peak 4 weeks after birth but they are smaller than those from mature neurons, and preferentially reach axonal boutons already contacted by other spines, presumably from mature neurons (Toni et al., 2007; Trinchero et al., 2017). Interestingly, spines from newborn GCs may compete and displace synaptic contacts from mature neurons (Toni et al., 2007). Something similar occurs in the CA3 region, where mossy fiber boutons from new neurons share thorny excrescences or spines with boutons from mature cells. With time, mossy fiber boutons associated exclusively with one thorny excrescence appear, again suggesting that some competition between axonal boutons from mature and newborn neurons may exist (Toni et al., 2008). Relevantly, it has been suggested that this competition may interfere with preexisting memory circuits and contribute to forgetting (Frankland et al., 2013; Akers et al., 2014). Therefore, 4–8 week-old GCs are already connected to the hippocampal circuitry and play a specific role in memory codification.

Eight week-old newborn GCs are generally considered fully mature neurons, perfectly integrated into the hippocampal memory circuit, as they are almost morphologically and electrophysiologically indistinguishable from developmentally generated mature GCs (Figure 3D). Therefore, 8 week-old GCs are not hyperexcitable (Mongiat et al., 2009; Dieni et al., 2013) and do not show enhanced synaptic plasticity (Ge et al., 2007; Gu et al., 2012). Nevertheless, newly generated GCs maintain certain particularities compared to developmentally born GCs. Importantly, the inputs from PER and EC to newborn GCs increase, at least, until 12 weeks after the birth of the cells (Vivar et al., 2012). Accordingly, these adult-born GCs still present higher structural plasticity and subtle morphological differences when compared with developmentally generated ones (Lemaire et al., 2012; Kerloch et al., 2018). For instance, memory tasks (Morris Water Maze) increase the complexity and number of spines of the dendritic trees of 8–16 week-old GCs but not developmentally generated GCs in rats (Lemaire et al., 2012). Hence, 8 week-old GCs are fully connected to the hippocampal circuitry and show electrophysiological mature properties while they retain higher structural plasticity compared to developmentally generated GCs.

### Functional Relevance of the Integration of Adult-Born GCs Into Memory Circuits

As we have discussed above, the integration of maturing GCs into the hippocampal circuitry is a slow and continuous process that generates a population of immature neurons in different stages with a range of functional characteristics: from almost silent, passing through a stage of hyperexcitability, to finally become rarely recruited by the incoming inputs (Piatti et al., 2013; Toda et al., 2018). Cells at each different maturation state may contribute to the hippocampal circuitry in particular ways: regulating feedback inhibition of mature GCs (Piatti et al., 2013), rewiring pre-existing connections (Goodwin, 2018), or establishing different memory codes for subtly different incoming signals (pattern separation) (McAvoy et al., 2015; Toda and Gage, 2018). However, the specific contribution of newborn neurons to hippocampal memory codification is a matter of an ongoing and exciting debate (Nakashiba et al., 2012; Frankland et al., 2013; Piatti et al., 2013; McAvoy et al., 2015; França, 2018; Toda et al., 2018).

### Regulation of Maturing GCs Integration Into Memory Circuits

Importantly, the involvement of maturing GCs in memory function indicates that memory may be affected by those factors that regulate the integration of these cells into the hippocampus, such as neuronal activity, which is initially sensed by neuroblasts in the form of tonic GABA and later in the form of inputs from inhibitory and excitatory local circuits (Ge et al., 2006; Jagasia et al., 2009; Song et al., 2013; Alvarez et al., 2016). In addition to surrounding neurons, other resident cells of the hippocampus may play a role in the integration of maturing GCs. This is the case of astrocytes, which have been shown to actively contribute to the incorporation of maturing GCs to the hippocampal circuit (Krzisch et al., 2015; Sultan et al., 2015). Microglia, the immune cells of the CNS, are also well suited to participate in the wiring of these adult-born cells, as they have been described to participate in the integration of newborn neurons into brain circuits during development, and in the activity dependent modification and pathological modulation of neuronal connectivity. In the next sections we will briefly introduce microglia and then review their role in the wiring of maturing neurons.

## Microglia

Microglia survey the brain parenchyma to maintain brain homeostasis, sensing changes in their environment, removing pathogens and cell debris by phagocytosis, undergoing changes in gene expression and morphology, and mounting the innate immune response when necessary (Sierra et al., 2014). Microglia present specific characteristics that make them unique and clearly distinct from other tissue macrophages. First, microglia have a different embryonic origin when compared with the majority of resident macrophages, as they originate from a myeloid progenitor cell derived from the yolk sac during early embryonic development (Ginhoux et al., 2010). Second, the population of adult microglia does not depend on the supply of peripheral precursor cells (blood monocytes), as it occurs with other adult tissue macrophages (Röszer and Röszer, 2018), but slowly renews itself through adulthood in humans and rodents (Askew et al., 2017; Réu et al., 2017), allowing them a certain degree of “cellular memory” of past events (Cheray and Joseph, 2018). Third, microglial processes are highly motile, and are able to scan the entire brain parenchyma every few hours while their cell body remains immobile (Nimmerjahn et al., 2005; Paris et al., 2018).

Microglia are very dynamic cells with extraordinary functional and morphological diversity. Accordingly, several single cell RNASeq studies have shown that microglia present a great variety of transcriptional responses that are far more complex than the polarization into M1 (pro-inflammatory or classic) and M2 (anti-inflammatory or alternative) states (Chiu et al., 2013; Xue et al., 2014; Gosselin et al., 2017). There is growing evidence that tissue-specific factors from local microenvironment dictate the functional states of developing and adult tissue-resident macrophages. Microglia express a large number of surface receptors, their “sensome,” which allows them to sense signals from other cells and the state of their environment, and to detect subtle changes induced by life-style factors, such as cognitive stimulation, physical exercise, diet or stress (Kettenmann et al., 2011; Hickman et al., 2013; Valero et al., 2016; Hanamsagar and Bilbo, 2017). In addition, while surveilling the parenchyma microglia communicate with other cells of the CNS and affect their function (Kettenmann et al., 2013). Some of the sensome receptors contribute to the direct communication between neurons and microglia, such as the receptor of the neuronal chemokine fractalkine (CX3CR1); purinergic receptors (e.g., P2X4, P2Y1, or P2Y12); triggering receptor expressed on myeloid cells 2 (TREM2) and its adaptor DNAX activation protein of 12KDa (DAP12), which participate in phagocytosis; or the CR3 receptor of the immune complement system (Pascual et al., 2012; Schafer et al., 2012; Scianni et al., 2013; Hickman et al., 2013; Eyo et al., 2014; Paolicelli et al., 2014). Their sensome allows microglia to adapt to changing environments and to mount an appropriate response. This communication with other cells allows microglia to shape the CNS playing a role in different developmental relevant processes such as blood vessel formation, myelination, and neurogenesis (Frost and Schafer, 2016).

In the next section we will review the current knowledge about the role of microglia in neurogenesis, focusing on their participation in the integration of maturing GCs into the hippocampal circuitry.

## Microglial Contribution to the Integration of Adult Generated Granule Cells Into Hippocampal Circuitry

### Microglial Participation in Adult Neurogenesis

Microglia invade the brain during embryonic development and their number peaks postnatally. In the mouse hippocampus microglial density reaches a maximum around postnatal day 15 (Paolicelli et al., 2011), suggesting that they may influence hippocampal neurogenesis since early postnatal days. In the adult neurogenic niche, microglia establish close contacts with all cells in the cascade, from rNSCs to neuroblasts and newborn neurons in physiological conditions (Sierra et al., 2010). Microglia also eliminate apoptotic ANPs and neuroblasts through phagocytosis, maintaining the homeostasis of the neurogenic niche (Sierra et al., 2010). Moreover, microglia may modulate neurogenesis through the secretion of different factors (e.g., cytokines, trophic factors, etc.) that have been suggested to affect proliferation, differentiation, and survival of newborn cells. Importantly, changes in microglia induced by neuroinflammation, aging, and pathology may contribute to the reduction in hippocampal adult neurogenesis and to the concomitant defects in memory function (Valero et al., 2012, 2016, 2017; Sierra et al., 2014). However, there are few data about the possible involvement of microglia on the integration of adult generated GCs into hippocampal circuits. Fortunately, the involvement of microglia in neuronal maturation and integration into brain circuits during development and pathology suggests that they may also participate in the integration of adult generated GCs into hippocampal circuits. We will now review the two major mechanisms by which microglia modulate neuronal integration: direct microglia-neuron interaction and the release of soluble factors (Table 1).

**Table 1:** Summary table.

Mechanism	Model	Reference
**Neurite growth**		
Microglia-neuron interactions	*In vivo*	Squarzoni et al., 2014; Baalman et al., 2015; Clark et al., 2016; Weinhard et al., 2018
	*Ex vivo*	Weinhard et al., 2018
Release of soluble factors	*In vivo*	Guthrie et al., 1995; Batchelor et al., 1999; Batchelor et al., 2002; Liu et al., 2017
	*Ex vivo*	Liu et al., 2017
	*In vitro*	Nagata et al., 1993; Huang et al., 2017
Unknown	*In vivo*	Bolós et al., 2017; Appel et al., 2018
**Structural synaptic plasticity**		
Microglia-neuron interactions	*In vivo*	Wake et al., 2009; Tremblay et al., 2010; Paolicelli et al., 2011; Schafer et al., 2012; Parkhurst et al., 2013; Chen et al., 2014; Kim et al., 2017; Lowery et al., 2017; Schecter et al., 2017; Filipello et al., 2018; Weinhard et al., 2018
	*Ex vivo*	Weinhard et al., 2018
Release of soluble factors	*In vivo*	Parkhurst et al., 2013
	*In vitro*	Lim et al., 2013
Unknown	*In vivo*	Bolós et al., 2017; Appel et al., 2018
**Functional maturation of synapses**		
Release of soluble factors	*In vivo*	Antonucci et al., 2012; Ferrini et al., 2013; Parkhurst et al., 2013; Liu et al., 2017
	*Ex vivo*	Pascual et al., 2012; Scianni et al., 2013; Zhang et al., 2014
	*In vitro*	Antonucci et al., 2012; Pascual et al., 2012; Scianni et al., 2013; Gabrielli et al., 2015; Liu et al., 2017
Unknown	*In vivo*	Roumier et al., 2004, 2008; Hoshiko et al., 2012


*Ex vivo, organotypic and acute slices; *In vitro*, cell cultures; *In vivo*, fixed tissue and life-imaging experiments*.

### Microglia-Neuron Interaction

Microglial motile processes continuously scan the brain parenchyma, establishing close contacts with neurites and synaptic regions (Kettenmann et al., 2013). While monitoring neurites and synapses, microglia may sense their state through the binding of their receptors to neuronal ligands. The interaction (or lack of thereof) of microglial receptors with neuronal ligands, such as fractalkine or the complement system molecules C1q/C3, induce specific responses in microglia that regulate their activity and motility (Kettenmann et al., 2013; Szepesi et al., 2018). However, the functional significance of this monitoring is still widely discussed, but has been suggested to contribute to neuronal connectivity by four mechanisms.

First, microglia are able to engulf small portions of axons, a process named trogocytosis, and contribute to limit axonal outgrowth and to eliminate presynaptic regions (Weinhard et al., 2018). Microglia have been also proposed to phagocytose dendritic spines (Paolicelli et al., 2011; Kim et al., 2017; Filipello et al., 2018); although recent evidence suggests that microglia only partially engulf spines, at least in the hippocampus (Weinhard et al., 2018), as it will be discussed in the next sections. Second, microglia may modulate neuronal connectivity by physically interposing their cell bodies and processes between post and presynaptic regions (Chen et al., 2014). Third, microglial-neuron interaction has been associated with the generation of filopodia from the dendritic shaft and the head of dendritic spines, contributing to the formation and relocation of dendritic spines, respectively (Miyamoto et al., 2016; Weinhard et al., 2018). Finally, microglia also establish close contacts with the axon initial segment (Baalman et al., 2015; Clark et al., 2016), suggesting a yet unexplored mechanism to affect neuronal connectivity. Therefore, through the establishment of close contacts with neurites and synaptic elements microglia participates in the elimination, interruption, and formation of neuronal connections.

### Release of Soluble Factors

In addition to cell-to-cell contact, microglia modulate neuronal wiring through the release of soluble factors. The microglial secretome contains several molecules, such as components of the extracellular matrix, trophic factors, cytokines, endocannabinoids, and microRNAs (miRs) that have been described to influence neuronal connectivity by affecting neurite growth, and structural and functional synaptic plasticity. Importantly, microglia may release these factors either anchored to the surface or encapsulated into extracellular vesicles (EVs) (Szepesi et al., 2018). The effect of these factors may be dose dependent as it occurs with the tumor necrosis factor α (TNFα), which is neuroprotective at low levels but exerts a pro-apoptotic activity at higher concentrations (Bernardino et al., 2008). Additionally, these factors have been postulated to induce effects both at long-distance, and locally while microglial processes are in close proximity to neurons, exerting a global or localized action in brain connectivity (Szepesi et al., 2018).

The data presented here suggest that microglia may modulate the connection of adult-born GCs to the hippocampal circuitry. However, few studies have specifically addressed the role of microglia in the wiring of adult-born neurons. In the next sections we will review the current knowledge about the role of microglia on neuronal wiring during development, activity-dependent synaptic remodeling and pathology by microglia-neuron contact as well as by release of soluble factors. We will focus in three processes that are relevant for neuronal integration into brain circuitry: neurite growth and maintenance, structural synaptic plasticity (formation, elimination, and relocation of synapses), and functional synaptic plasticity (functional molecular changes in pre- and postsynaptic compartments). Finally, at the end of each section we will discuss the role of microglia in the integration of adult-generated GCs into the hippocampal circuitry.

## Microglial Modulation of Neurite Growth and Maintenance

Neurite growth involves the initiation, enlargement (including branching), and final stabilization of both dendrites and axons. Neurite outgrowth is highly relevant for adult-born GCs mainly 1–3 weeks after their birth, when these cells extend their dendrites and axons toward the ML and CA3/CA2, respectively. Nevertheless, adult-born GCs may retain the capacity of increasing their dendritic tree upon cognitive stimulation even 16 weeks after their birth (Lemaire et al., 2012).

Direct evidence shows that microglia modulate developmental neurite growth, as their constitutive ablation (by knocking out the transcription factor PU.1) or in pathological conditions (through conditioned expression of the diphtheria toxin receptor in these cells and subsequent administration of the diphtheria toxin) affects both axon sprouting in the striatum and neurite length and complexity in the hippocampus and spinal cord (Squarzoni et al., 2014; Liu et al., 2017). Importantly, the role of microglia in neurite growth differs between different regions of the CNS, as microglia do not participate in the regeneration of retinal ganglion axons after optical nerve crush (Hilla et al., 2017), while they contribute to decrease the complexity of hippocampal dendrites and to increase the complexity of spinal cord dendrites after spinal cord injury (Liu et al., 2017). The effects caused by microglial depletion in neurites during development and in pathological conditions suggest that they may participate in the dendritic and axonal sprouting of newborn GCs that occurs since the early stages of their maturation. In the next sections we will review the different mechanisms proposed for the modulation of neurite growth by microglia and finally discuss whether they may be involved in the integration of adult-born GCs.

### Microglia-Neuron Interaction

Microglia may modulate axonal growth through direct contact with axonal regions. During embryonic and postnatal development in the mouse striatum microglia establish close contacts with dopaminergic axons and engulf them, suggesting that they may limit axonal growth by actively remove axonal fragments (Squarzoni et al., 2014). Direct evidence of microglial engulfment of small axonal regions (trogocytosis) was observed using live imaging in postnatal hippocampal slices and verified using 3D electron microscopy reconstructions, although their contribution to axonal sprouting was not analyzed (Weinhard et al., 2018). In addition, microglia may also contribute to axonal maintenance through physical contact with the axon initial segment of, mainly, excitatory neurons. The interaction of microglia with the axon initial segment was observed both during postnatal development and adulthood; is more prominent in the mouse cortex than in other regions of the brain, such as the thalamus and the striatum; and is preserved across species (mouse, rats and non-human primates) (Baalman et al., 2015). It has been speculated that this contact of microglia with the axon initial segment may provide trophic support. However, data are contradictory as in a model of traumatic brain injury microglial contacts with initial segments diminish suggesting loss of trophic support, while in the experimental model of demyelination by cuprizone and chronic autoimmune encephalomyelitis microglial contacts with axon initial segments increase and precede their axonal breakdown, suggesting that microglial contacts are detrimental for axonal integrity (Baalman et al., 2015; Clark et al., 2016). Therefore, microglial close contact with neurites may restrict their growth through trogocytosis, and be involved in their maintenance.

### Release of Soluble Factors

Microglia have been shown to regulate neurite growth through the release of different soluble factors. First, microglia release components of the extracellular matrix such as plasminogen and thrombospondin, which *in vitro* induce neurite outgrowth (Nagata et al., 1993; Chamak et al., 1994, 1995). Second, several studies indicate that microglia induce neurite growth by releasing different factors after injury such as brain derived neurotrophic factor (BDNF) in the striatum, insulin growth factor-1 (IGF-1) in the hippocampus, and TNF-α in the spinal cord and hippocampus (Guthrie et al., 1995; Batchelor et al., 1999; Batchelor et al., 2002; Liu et al., 2017). TNF-α deserves special attention, as it has been argued to be exclusively expressed by microglia in the CNS (Barres, 2008) and to meditate the effects induced by spinal cord injury in the decrease and increase of the dendrites of hippocampal and spinal cord neurons of mice, respectively (Liu et al., 2017). Accordingly, TNF-α affects neuronal branching *in vitro* in a dose dependent manner. Thus, low levels of TNF-α increase neuronal branching in mouse postnatal SVZ neurospheres, while higher doses have no effects in neurospheres or reduce the branching of cultured neurons from the hippocampus of rat embryos (Bernardino et al., 2008; Keohane et al., 2010). Finally, microglia may affect neurite growth through the release of EVs carrying modulatory molecules; this is the case for pre-micro RNA miR-124-3p, which is released via exosomes by the microglial cell line BV2 (Huang et al., 2017). BV2 cells treated with brain extracts from experimental mouse models of traumatic brain injury secrete exosomes enriched in miR-124-3p that, *in vitro*, induce neurite outgrowth (Huang et al., 2017). Importantly, miR-124 is also found in microglia acutely purified from the adult brain (Ponomarev et al., 2011) although its activity is exclusively detected in neurons (Åkerblom and Jakobsson, 2014). Thus, it is possible that the inactive form of miR-124 may be released by microglia encapsulated in exosomes, reach neurons, and then become activated promoting neurite outgrowth, although this mechanism remains to be demonstrated. Hence, microglia release different factors that have been demonstrated to contribute to the neurite changes that occur during development and in pathological conditions.

### Microglial Modulation of Neurite Growth in Adult Neurogenesis

**FIGURE 4:**
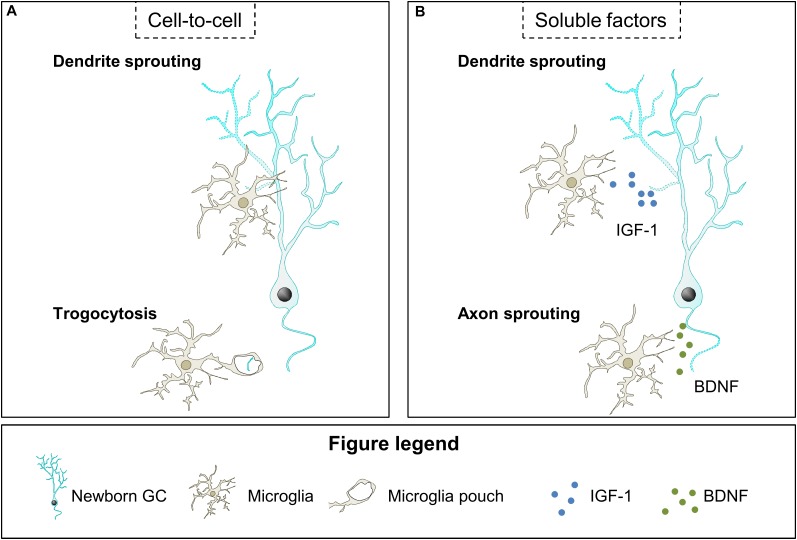
Hypothetical contribution of microglia to neurite growth. **(A)** Microglia contact newborn GC dendritic tree and may modulate dendrite sprouting through an unknown mechanism. Also, microglia may engulf small segments of the newborn GC axon and restrict neurite growth. **(B)** Microglia have been shown to secrete soluble factors that may contribute to dendrite and axon sprouting, such as IGF-1 and BDNF, respectively. BDNF, brain derived neurotrophic factor; IGF-1, insulin growth factor-1.

The described effects of microglia on neurite outgrowth during development and pathologies may be relevant in the context of adult neurogenesis. We speculate that microglial contacts may modulate neurite sprouting in maturing GCs during the first 3 weeks after their birth, when these cells extend their dendrites toward the ML and axons toward the CA3/CA2 regions; or affect axonal integrity afterward, once initial neurite growth has concluded (Figure 4A,B).

Importantly, several studies using mouse models in which microglia are dysfunctional have suggested their involvement in dendritic tree sprouting of adult-born GCs (Figure 4A). In these studies both mice with microglial-specific depletion of the vacuolar sorting protein 35 (VSP35), involved in endosomal trafficking to the Golgi, and mice lacking CX3CR1 show a reduction in length and complexity of newborn GCs dendritic tree and concomitant alterations in microglial morphology and density (Rogers et al., 2011; Pagani et al., 2015; Bolós et al., 2017; Appel et al., 2018). Although these data indicate that microglia play a role in neurite growth of maturing GCs the underlying mechanisms have not been explored. These effects may be mediated by direct microglia-neuron contact, as microglia establish stable and transient contacts with the dendrites of 3–7 week-old newborn GCs (Chugh and Ekdahl, 2016). The functional relevance of these contacts is unknown and they may modulate dendritic sprouting but also the dynamics of dendritic spines, as we will review in the next section. In addition, microglia may also control axonal sprouting through trogocytosis of newborn mossy fiber axons on their way to CA3, a process in which microglia have been involved during development (Squarzoni et al., 2014; Weinhard et al., 2018). Although there is no evidence of the effects of the close contacts between microglia and neurites of maturing GCs, there are some studies indicating that microglia may act on neurite sprouting of maturing GCs by releasing soluble factors, as it will be next discussed.

Microglial action on neurite growth of maturing GCs may also be mediated by local release of soluble factors such as IGF-1 and BDNF (Figure 4B). IGF-1 and BDNF are well-known promoters of neurite growth (Bondy and Cheng, 2004; Guo et al., 2018) and they are overexpressed by microglia after voluntary exercise (Kohman et al., 2012; Littlefield et al., 2015), which is known to increase the complexity of the dendrites of newborn GCs in mice (Dostes et al., 2016; Sah et al., 2017; Trinchero et al., 2017). However, in both normal and voluntary exercise conditions, microglial BDNF effects on dendritic sprouting of maturing GCs are probably small, as it mainly depends on the autocrine production of BDNF (Wang et al., 2015). Interestingly, in the same study lack of autocrine secretion of BDNF did not affect axonal sprouting, which leaves open the possibility that microglial BDNF may act on axons. Indeed, it is plausible that microglial BDNF promotes axonal formation by binding to the neurotrophin receptor p75NTR, whose activation is necessary for the initiation of the axon of adult-born GCs (Zuccaro et al., 2014). Altogether, the data presented here indicate that microglia may be involved in the sprouting of newborn GCs neurites by contacting them and releasing neurotrophic factors under conditions such as voluntary exercise, although this possibility should be directly tested in future studies.

## Microglial Involvement in Structural Synaptic Plasticity

Structural synaptic plasticity involves changes in the morphology of pre- and postsynaptic elements: synapse elimination, formation, and remodeling. Newborn GCs transverse critical periods of structural synaptic plasticity from 2 to 8 weeks after birth (Zhao et al., 2006; Toni et al., 2007; Trinchero et al., 2017). Several factors, such as exercise and cognitive stimulation, potentiate the formation of dendritic spines; while others, such as aging, delay their formation (Tronel et al., 2010; Alvarez et al., 2016; Trinchero et al., 2017). Microglia may participate in this modulation of GCs structural synaptic dynamics; as they are able to sense life-related factors and contribute to structural synaptic plasticity during development, pathology, and physiological activation of neurons. Several pieces of evidence indicate that microglia exert their action on synaptic remodeling through the establishment of microglia-neuron contacts with synaptic elements and through the release of soluble factors, as we will review here. Finally, the possible role of microglia in structural synaptic plasticity of adult-born GCs will be discussed in the last part of this section.

### Microglia-Neuron Interaction

Microglia establish close contacts with pre- and postsynaptic regions that may result in their engulfment and elimination, the loss of synaptic contacts by physical interposition, and the formation of filopodia that induce the generation and/or relocation of dendritic spines. Here we will review previous studies suggesting the existence of these three possible mechanisms of action.

Microglia have a crucial role in the physiological removal of the excess of synapses produced during development, a phenomenon called “synaptic pruning” (reviewed in Kettenmann et al., 2013; Salter and Beggs, 2014; Mosser et al., 2017). In this regard, microglia have been described to be involved in the elimination of presynaptic and postsynaptic (dendritic spines) elements. For instance, visual cortex microglia contact dendritic spines in an activity-dependent manner, indicating that these contacts may be relevant for the function of the dendritic spines and their dynamics (Wake et al., 2009; Tremblay et al., 2010). The role of microglia in the elimination of dendritic spines was suggested by data showing that delayed microglial invasion of the mouse hippocampus, induced by the lack of CX3CR1, leads to delayed decrease in dendritic spines (Paolicelli et al., 2011). Furthermore, microglial processes have been shown to contain puncta positive for classical postsynaptic markers, such as the postsynaptic density protein 95, *in vitro* and *in vivo* in the mouse cortex and hippocampus (Paolicelli et al., 2011; Kim et al., 2017; Appel et al., 2018; Filipello et al., 2018). However, although microglial trogocytosis of axonal portions has been demonstrated, phagocytosis of spines has not been directly observed. Indeed, a recent study indicated that postsynaptic elements are not phagocytosed by microglia, at least in the postnatal (P15) hippocampus, where apparently engulfed dendritic spines are always found connected to the dendrite through the spine neck (Weinhard et al., 2018). Importantly, microglial contacts with synaptic elements are prominent during the peak of plasticity of the visual cortex (P28) and have been related to the elimination of synapses through engulfment of presynpatic but not postsynaptic regions, as CX3CR1 KO mice show a reduction in the number of microglial contacts with axon terminals and a concomitant increase in axonal density (Lowery et al., 2017; Schecter et al., 2017). Relevantly, microglia eliminate presynaptic elements in an activity-dependent manner in the P5 dorsal lateral geniculate nucleus (dLGN) of mice as reduced and increased activity of retinal ganglion cells (RGCs) potentiates and decreases, respectively, axon terminals engulfment by microglia (Schafer et al., 2012). In the dLGN, the complement receptor CR3 is necessary for microglia engulfment of axon terminals, as CR3 KO mice have increased axon density and decreased axon colocalization with microglial staining, suggesting decreased engulfment of axon terminals (Schafer et al., 2012). However, CR3 is involved in the elimination of presynaptic regions only in some regions of the brain as the hippocampus of CR3 KO mice shows similar levels of trogocytosis when compared with control mice (Weinhard et al., 2018). Therefore, the elimination of axonal terminals may be mediated by trogocytosis, while the mechanism of dendritic spine elimination is not known. We speculate that dendritic spines disappearance may be related to the lack of contact with a presynaptic terminal, which may be induced by both the uncompleted engulfment of the spine or the elimination of the presynaptic terminal performed by microglia.

In addition to engulfing synaptic regions, microglia interfere with synapses by physically interposing their cell body and processes between pre- and postsynaptic elements. This mechanism of synaptic interference has been described in inhibitory synapses in the mouse cortex after the induction of systemic inflammation by intraperitoneal administration of LPS (gram negative bacteria lipopolysaccharide), when microglia displace inhibitory synaptic contacts from the surface of the soma of pyramidal neurons (Chen et al., 2014). The microglial surrounding of the pyramidal neuron soma is speculated to decrease inhibitory input and thus to increase neuronal firing and neuronal synchronicity (Chen et al., 2014). Additionally, the partial engulfment of dendritic spines described by Weinhard et al. in the postnatal mouse hippocampus may also dislodge excitatory pre- and postsynaptic regions (Weinhard et al., 2018), although this is highly speculative, as interruption of excitatory synapses by microglial processes has not been analyzed properly. These data indicate that microglial direct interposition between pre- and postsynaptic elements may interfere with synapses.

Finally, the microglial close contact with neurons may lead to the formation and/or relocation of dendritic spines during postnatal development by promoting the growth of dendritic shaft or spine head filopodia. Direct evidence of the role of microglia in dendritic spines formation comes from their genetic depletion by diphtheria toxin mice in the early postnatal barrel cortex and the adult motor cortex (Parkhurst et al., 2013; Miyamoto et al., 2016). Importantly, Miyamoto et al., indicated that microglia promote dendritic spine formation by contacting dendrites and propitiating the generation of dendritic shaft filopodia (early stage dendritic spines) (Yoshihara et al., 2009), presumably through the induction of transient local rises in calcium levels and subsequent actin polymerization in the contacted region (Miyamoto et al., 2016). Spine head filopodia induction by microglia has also been proposed to relocate dendritic spines in the hippocampus of P15 mice, as their contacts with the head of hippocampal dendritic spines is followed by the formation of spine head filopodia and the relocation of the dendritic spines (Weinhard et al., 2018). In summary, previous studies indicate that contact of microglia with dendrites during postnatal development contributes to the remodeling of synapses through the induction of elimination of dendritic spines and trogocytosis of presynaptic regions, the interruption of synaptic communication, and the formation and relocation of dendritic spines by filopodia.

### Release of Soluble Factors

Microglia release several soluble factors which may contribute to the formation and elimination of pre- and postsynaptic terminals. One key factor seems to be BDNF, a well-known pro-survival factor controlling synaptic plasticity (Leal et al., 2014). BDNF is required for the formation of spine head filopodia in dendritic spines and the relocation of spines to multisynaptic boutons in the mouse hippocampus and olfactory bulb *in vivo* (Breton-Provencher et al., 2016; Niculescu et al., 2018), although the cellular origin of this BDNF is unclear. Importantly, microglia BDNF contributes to activity-dependent formation of dendritic spines in the motor cortex (Parkhurst et al., 2013), possibly by inducing spine head filopodia emerging from the dendritic shafts (Miyamoto et al., 2016) although the actual mechanism remains unknown. Another soluble microglial mediator involved in synaptic structural plasticity is interleukin-10 (IL-10), which increases the number of pre- and postsynaptic terminals in neuronal cell cultures from rat hippocampus, an effect that is antagonized by interleukin-1β (IL-1β) (Lim et al., 2013). TNF-α may also contribute to dendritic spine remodeling, at least in pathological conditions, as after spinal cord injury TNF-α contributes to the decrease and increase of dendritic spines in the hippocampus and spinal cord, respectively (Liu et al., 2017). Therefore, microglia release several soluble factors that modulate structural synaptic plasticity.

### Microglial Modulation of Structural Synaptic Plasticity in Adult Neurogenesis

**FIGURE 5:**
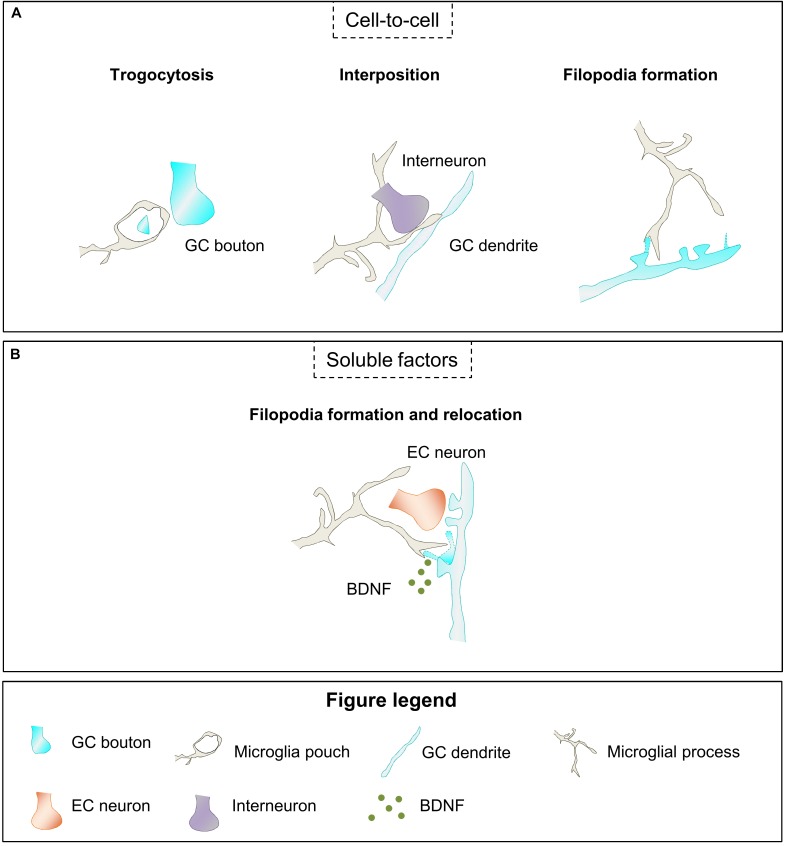
Hypothetical contribution of microglia to structural synaptic plasticity of newborn GCs. **(A)** Microglia may modulate synaptic structural plasticity by eliminating small parts of the GC bouton (trogocytosis), interfering with GABAergic synapses (interposition), and inducing the formation of new filopodia in GC dendrites or spines. **(B)** Microglia secrete BDNF which may participate in filopodia formation and relocation to multisynaptic boutons. BDNF, brain derived neurotrophic factor; EC, entorhinal cortex; GC, granule cell.

The data above suggest that microglia may exert a role on structural synaptic plasticity of newborn GCs. Thus, microglia may participate in the formation, relocation and elimination of the excess of synaptic contacts established by maturing GCs from 2 to 8 weeks after birth, when newborn GCs transverse critical periods of structural synaptic remodeling. Microglia may act using several of the mechanisms described above based in close microglia-neuron contact (elimination, interposition, or formation and relocation of synaptic contacts) or the release of soluble factors (Figure 5A,B).

Microglia may modulate maturing GCs connectivity through the elimination of pre- and postsynaptic regions. Although this hypothesis has not been directly analyzed, a recent study suggested that microglia may regulate the shape of axon terminals of maturing GCs in mice, as lack of CX3CR1 decreased their area (Bolós et al., 2017). Microglia may also interfere with synaptic regions avoiding synaptic communication. Indeed, microglia may interfere with the GABAergic input received by maturing GCs, as it occurs with inhibitory synapses in the mouse cortex after systemic inflammation induction by intraperitoneal administration of LPS (Chen et al., 2014), suggesting that this interference may have relevant and different consequences for maturing GCs depending on the time in which it occurs. Therefore, microglial interference with GABAergic synapses might decrease maturation and survival of maturing GCs if it takes place when GABA is excitatory (until 4 weeks after their birth); or decrease the inhibitory input into GCs when GABA inhibits them (before 4 weeks after their birth), thus contributing to their higher excitability. It should be taken into account that the microglial interference with GCs GABAergic input is highly speculative and, as far as we know, there are no studies analyzing this hypothesis. Another possibility is that microglia contribute to the formation and relocation of dendritic spines from 2 to 8 week-old maturing GCs. Data from previous studies suggested that microglia are involved in structural synaptic remodeling of maturing GCs but did not indicated whether they participate in synaptic elimination, formation, or both (Bolós et al., 2017; Appel et al., 2018). The density of dendritic spines of newborn GCs is reduced in the adult hippocampus of mice lacking microglial specific proteins relevant for their function such as the fractalkine receptor CX3CR1 (Bolós et al., 2017) or the VSP35 (Appel et al., 2018). These data suggest that microglia may regulate structural synaptic plasticity of maturing GCs through microglia-neuron interaction (Figure 5A).

Finally, although there is no direct evidence of microglial release of soluble factors that contribute to the dynamics of synapses in maturing GCs, the formation of spine head filopodia has been related to BDNF levels in adult-born neurons of the mouse olfactory bulb (Breton-Provencher et al., 2016), suggesting that BDNF may also exert a similar effect in adult-born hippocampal neurons (Figure 5B). We speculate that microglia may be the source of this BDNF, as microglial BDNF is known to contribute to the formation of dendritic spines (Parkhurst et al., 2013) in the mouse cortex; and microglia have been described to participate in the formation of dendritic spines by inducing the generation of dendritic shaft filopodia in the mouse somatosensory cortex (Miyamoto et al., 2016). Hence, microglia may contribute to the characteristic location of maturing GCs dendritic spines to multisynaptic boutons (Toni et al., 2007) by inducing the formation of spine head filopodia, which has been related to the relocation of dendritic spines to multisynaptic regions in the postnatal mouse hippocampus (Weinhard et al., 2018). While there is no direct evidence for this hypothesis, we suggest that microglia may act in GCs synaptic regions by establishing close contacts with them to induce their elimination, interrupt synaptic communication, or contribute to their formation and relocation; and by releasing soluble factors such as BDNF. Altogether, these data point toward a possible role of microglia during the development of synaptic contacts that integrate adult-born GCs into the hippocampal circuitry.

## Microglial Regulation of Functional Maturation of Synapses

Microglia may exert a role in the functional maturation of synapses through the release of soluble factors that induce changes in the molecular composition of pre- and postsynaptic compartments (microglia-neuron contact has not been involved in the functional maturation of synapses). These mechanisms are relevant for the integration of newborn GCs into the hippocampal circuitry (2–4 weeks after their birth), and their recruitment to memory engrams and final maturation (4–8 weeks after their birth). Therefore, we will discuss the participation of microglia on functional synaptic maturation of adult-born GCs by modulation of GABA-mediated inhibition and excitatory synaptic efficacy during development and pathology.

### Microglial Modulation of GABA-Mediated Inhibition

Microglia may modulate GABA mediated inhibition through the release of soluble factors that may affect pre- and postsynaptic terminals. For instance, microglial BDNF modulates the inhibitory effect of GABA in neurons by affecting the expression of the contransporter KCC2 (Ferrini and De Koninck, 2013; Ferrini et al., 2013). KCC2 normally increases during neuronal maturation, contributing to the change in the action of GABA from excitatory to inhibitory in maturing neurons (Rivera et al., 2005). Evidence of the role of microglia in the modulation of KCC2 expression comes from animal models of pathological pain, in which microglial BDNF was shown to bind to TrkB receptor expressed by neurons, promoting its phosphorylation, and triggering a decrease in KCC2, which subsequently reduces GABA mediated inhibition, thus sensitizing and inducing pathological pain (Ferrini and De Koninck, 2013). In these models of pathological pain, microglial BDNF release is induced by ATP acting on microglial P2X4, which is nonetheless expressed at very low levels in physiological conditions (Ferrini and De Koninck, 2013; Ferrini et al., 2013). In addition to affect GABA sensing in the postsynaptic region, microglia may also affect presynaptic transmission in GABAergic neurons by releasing EVs. Rat primary microglia exposed to ATP release EVs that carry on their surface the endocannabinoid N-arachidonoylethanolamine (AEA), which *in vitro* activates CB1 cannabinoid receptors of GABAergic neurons decreasing inhibitory transmission (Gabrielli et al., 2015). Therefore, microglia may modulate GABAergic transmission acting in the postsynaptic element through the release of BDNF and in the presynaptic region through the activation of the endocannabinoid system.

### Microglial Modulation of Excitatory Synaptic Efficacy

Microglia have been suggested to participate in functional synaptic maturation through the modulation of excitatory synaptic efficacy. We will review in this section the role of microglia in the modulation of postsynaptic regions through their influence in AMPA/NMDA ratio and GluN2B content of NMDA receptors. In addition, microglia collaborate with astrocytes to potentiate glutamatergic transmission through a mechanism that involves adenosine or ATP release by microglia, after fractalkine or LPS action, respectively (Pascual et al., 2012; Scianni et al., 2013). However the microglia-astrocytes crosstalk is not strictly considered a mechanism of synaptic functional plasticity and it is out of the scope of this review. Finally, although data about microglial effect on the composition of the presynaptic regions is scarce we will also briefly discuss this possibility.

Microglia have been involved in the control of synaptic efficacy through the modulation of relative levels of activity of AMPA and NMDA receptors at postsynaptic regions. The increase in synaptic AMPA receptors is relevant for synapse transformation from silent into functional, and to increase synapse efficacy (Petralia et al., 1999; Renger et al., 2001). Intact microglial function is required for the adequate control of the postnatal rise in AMPA/NMDA ratio, as it is abolished in the barrel cortex of P9 mice lacking CX3CR1 expression (Hoshiko et al., 2012) while it is increased in the hippocampus of P18-25 mice lacking or wearing a non-functional mutation of DAP12, specifically expressed by microglia in the brain (Roumier et al., 2004, 2008). In addition, microglia may affect AMPA/NMDA ratio in opposite directions in different regions of the CNS. Evidence arises from a mouse model of spinal cord injury in which microglia are required for the increase in AMPA/NMDA ratio occurring in the hippocampus after injury, and for the decrease of this ratio in the spinal cord, effects that are suggested to be mediated by microglial TNF-α (Liu et al., 2017). Finally, microglia may induce AMPA receptor endocytosis in postsynaptic terminals of hippocampal slices through the production of superoxide after simultaneous exposition to hypoxia and LPS, a mechanism that is dependent in CR3 activation and that induced long term depression (Zhang et al., 2014). These data indicate that microglia modulate postnatal and pathological changes in AMPA/NMDA ratio.

Another mechanism by which microglia may influence excitatory synaptic efficacy is by affecting the composition of NMDA receptors. The implication of microglia in functional maturation of postsynaptic sites is suggested by the analysis of mice with dysfunctional microglia due to the lack of DAP12 or CX3CR1. The brain of these mice shows increased proportion of the GluN2B subunit of NMDA receptors (Roumier et al., 2004, 2008; Hoshiko et al., 2012). In CX3CR1 KO mice this effect is transient and coincides with a time of delayed migration of microglia into the barrel cortex, suggesting that the presence of microglia is required for synaptic maturation (Hoshiko et al., 2012). Importantly, microglia may exert a different effect in the composition of extra-synaptic and synaptic NMDA receptors, as genetic depletion of microglia using the diphtheria toxin leads to increased levels of GluN2B in the whole brain but decreases levels in synaptosomes (Parkhurst et al., 2013). These data imply that microglia may increase the proportion of synaptic immature NMDA receptors, i.e., those containing GluN2B instead of GluN2A subunits, and thus increase the strength of the synapses (for further information about the role of GluN2B in synapses check: Shipton and Paulsen, 2014; Volianskis et al., 2015). Importantly, microglial BDNF is required for functional maturation of synapses, as mice lacking microglial expression of BDNF also show decreased levels of synaptic GluN2B (Parkhurst et al., 2013). Therefore, microglia may be involved in the modulation of the efficacy of synapses through their influence in synaptic levels of GluN2B containing NMDA receptors.

While the data above show that microglia modulates functional plasticity of postsynaptic regions, their involvement in the maturation of presynaptic regions is not well known. In the diphtheria toxin mouse model, depletion of microglia or lack of microglial BDNF leads to decreased levels of the presynaptic protein vesicular glutamate transporter 1 (vGlut1), suggesting alterations in the presynaptic compartment that may be related to a decrease in glutamate release (Parkhurst et al., 2013). In addition, microglial released EVs may also increase presynaptic glutamatergic transmission through the induction of sphingosine synthesis, which favors exocytosis and subsequent glutamate release, as it has been observed in primary neuronal cell cultures and the rat visual cortex (Antonucci et al., 2012; Gabrielli et al., 2015). Although further analyses are required to understand the involvement of microglia in functional maturation of postsynaptic regions, current data indicate that microglial release soluble factors such as BDNF, TNF-α, and EVs, that modulate functional synaptic maturation of both pre- and postsynaptic elements.

### Microglial Modulation of Functional Synaptic Plasticity in Adult Neurogenesis

**FIGURE 6:**
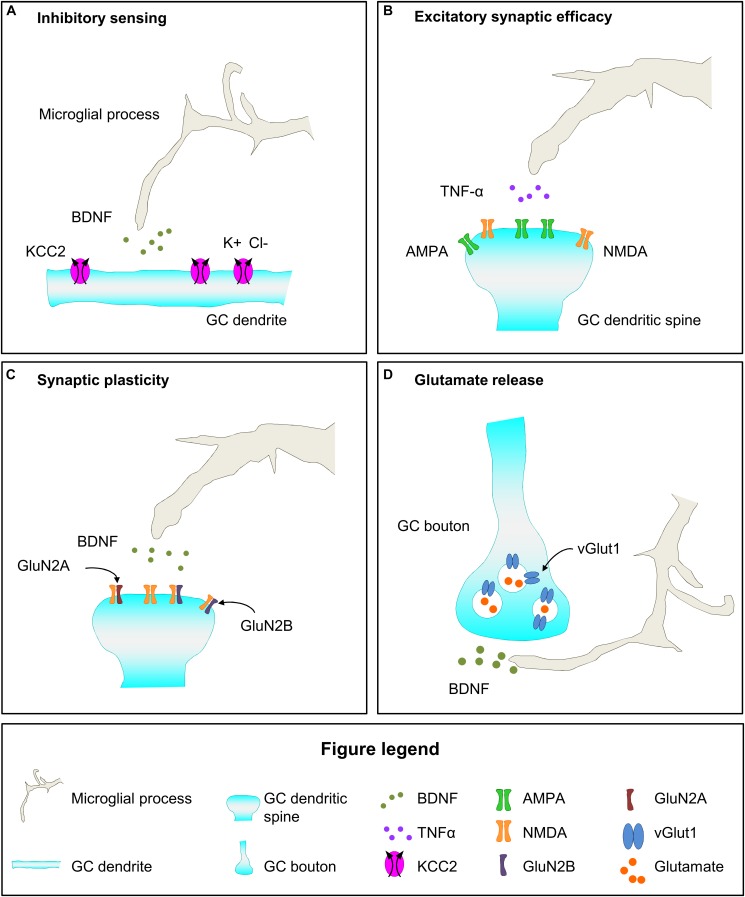
Hypothetical mechanisms of microglial regulation of functional maturation of synapses of newborn GCs. **(A)** Microglia may secrete BDNF, which upregulates the expression of the cotransporter KCC2 in newborn GCs, and increases their inhibitory sensitivity to GABA. **(B)** Microglia derived TNF-α may increase synaptic efficacy by increasing the AMPA/NMDA ratio. **(C)** Microglial BDNF may contribute to the enhanced synaptic plasticity of newborn GCs by raising the proportion of GluN2B subunit in NMDA receptors. **(D)** Microglia secreted BDNF may increase glutamatergic transmission by upregulating the expression of vGlut1 in presynaptic vesicles. BDNF, brain derived neurotrophic factor; Cl, chloride; GC, granule cell; K, potassium; TNF-α, tumor necrosis factor-α; vGlut1, vesicular glutamate transporter 1.

The data discussed above indicate that microglia participate in different aspects of functional synaptic maturation in development and pathological conditions, and thus suggest that they may contribute to functional synaptic maturation of newborn GCs. First, microglia may affect the excitatory and inhibitory effect of GABA in maturing GCs as indicated by the role of microglial BDNF release on neuronal KCC2 content in pathological conditions (Figure 6A; Ferrini and De Koninck, 2013; Ferrini et al., 2013). Importantly, final maturation of newborn GCs involves the increase in the expression of the KCC2 transporter, which is necessary to sense GABA as inhibitory, a fundamental change in GCs maturation (Ge et al., 2006). Second, microglia may participate in the maturation of glutamatergic excitatory contacts of adult-born GCs. A relevant step of GCs maturation is the incorporation of AMPA receptors to excitatory synapses to make them functional and increase their efficacy, a process in which microglia have been involved during development (Figure 6B; Roumier et al., 2004, 2008; Hoshiko et al., 2012). Furthermore, microglia may participate in the maintenance of the enhanced synaptic plasticity that defines 4–8 weeks maturing GCs by releasing BDNF and contributing to the high content in GluN2B that characterize these cells (Figure 6C; Schmidt-Hieber et al., 2004; Ge et al., 2007; Gu et al., 2012; Parkhurst et al., 2013). Regarding the presynaptic compartment, microglial BDNF may favor glutamate release by increasing vGlut1 levels in mossy fiber presynaptic vesicles (Figure 6D), as it has been shown during development (Parkhurst et al., 2013). Microglia have been demonstrated to modulate functional synaptic maturation in pathological conditions and during development, leading to the provocative hypothesis that microglia are involved in the functional maturation of adult-born GC, ultimately impacting on memory function.

## Conclusion

The integration of maturing GCs into the adult hippocampus is a continuous process that generates a range of immature neurons with different structural and functional characteristics. Young neurons at each different maturation state may contribute to the hippocampal circuitry in particular ways. Therefore, the study of the mechanisms involved in the integration of newborn GCs into the hippocampal circuit is necessary to fully understand memory function and its regulation. Several lines of evidence lead us to propose that microglia may participate in the integration of adult generated GCs into hippocampal memory circuits by controlling neurite growth, formation and elimination of synapses, and changing the molecular composition of synapses. The role of microglia in the wiring of newborn GCs may be particularly relevant for the effects that life factors and disease exert in the integration of newborn GCs into memory circuits, as microglia are able to detect subtle changes in their environment such as those induced by cognitive stimulation, physical exercise, diet, stress, inflammation or pathology (Valero et al., 2016, 2017). The development of experimental animal models that allow the depletion of microglia during a specific temporal window or the inhibition of the expression of soluble factors exclusively in microglia (e.g., BDNF) have been crucial to demonstrate the involvement of microglia in the incorporation of neurons into brain circuits. These mouse models offer an excellent opportunity to investigate the role of microglia in the incorporation of adult newborn GCs into hippocampal circuits and memory function. Elucidating the nature of the intricate relationships between microglia, integration of adult newborn GCs into the hippocampal circuit, and behavior is highly relevant to properly understand the contribution of microglia to memory function in physiological and pathological conditions.

## Author Contributions

NR-I created the schemes included in this manuscript. JV wrote the initial draft of the manuscript with input from all authors. AS and NR-I critically revised the manuscript. All authors reviewed and approved the manuscript.

## Conflict of Interest Statement

The authors declare that the research was conducted in the absence of any commercial or financial relationships that could be construed as a potential conflict of interest.
